# Ocular Biocompatibility of Poly-N-Isopropylacrylamide (pNIPAM)

**DOI:** 10.1155/2016/5356371

**Published:** 2016-11-01

**Authors:** Luiz H. Lima, Yael Morales, Thiago Cabral

**Affiliations:** ^1^Doheny Eye Institute, University of California, Los Angeles (UCLA), Los Angeles, CA, USA; ^2^Federal University of Sao Paulo (UNIFESP), Sao Paulo, SP, Brazil; ^3^Federal University of Espírito Santo (UFES), Vitória, Brazil

## Abstract

*Purpose.* To study the safety of intravitreal injections of poly-N-isopropylacrylamide (pNIPAM) tissue adhesive in rabbit eyes.* Methods.* Twelve study rabbits received an intravitreal injection of 0.1 mL 50% pNIPAM in the right eye. Follow-up examinations included color fundus photography, fundus fluorescein angiography (FA), optical coherence tomography (OCT), and electroretinography (ERG). Subsequent to the last follow-up assessment, the rabbits were sacrificed and histopathological study on the scleral incision sites was performed.* Results.* All study animals developed mild to moderate levels of inflammatory reaction in the conjunctiva, anterior chamber, and the anterior vitreous during the first month of follow-up. After this period, the level of the inflammatory reaction progressively decreased and completely disappeared after the third month of follow-up. The lens and cornea remained clear during the entire follow-up period. OCT and FA did not show areas of retinal damage or neovascularization. Histological and ERG studies of eyes injected with pNIPAM demonstrated absence of retinal toxicity.* Conclusion.* Intravitreal injections of pNIPAM were nontoxic in this animal study, and pNIPAM may be safe to be used as a bioadhesive in certain retinal diseases.

## 1. Introduction

Potential applications for tissue adhesive in ophthalmology surgery have generated growing interest. Adhesives are already being successfully used in the treatment of various anterior segments conditions [1–10]. Experimental models of rhegmatogenous retinal detachment and repair during vitreous surgery have been reported using transvitreal tissue adhesives such as cyanoacrylate, fibrin glue, and transforming growth factor-beta for retinopexy [11–15]. However, many of the tested adhesives elicited severe inflammation of the eye. Some proved to be toxic to the retina; others did not have sufficient adhesive strength. As most polymeric adhesives form very rapidly via a highly exothermic reaction, the heat and the chemical impurities discharged during the reaction can cause toxic damage and inflammatory response [16]. A safe and effective adhesive could potentially be used to manage retinal tears and treat macular holes and selected cases of retinal detachment (RD). However, an adhesive meeting these criteria for retinal tissue is not yet available for use.

Poly-N-Isopropylacrylamide (pNIPAM) is the most broadly studied thermoresponsive polymer for therapeutic targets. pNIPAM has reversible phase transition upon heat and inverse solubility. Below its lower critical solution temperature (LCST), that is, 32°C, it is hydrophilic and water soluble. However, above 32°C, it is hydrophobic and turns into a viscous gel which adheres to tissue [17]. pNIPAM has been already used for therapeutic purposes, for example, in drug targeting for solid tumors [18, 19], in drug delivery as a thermosensitive coating [20, 21], and in tissue culture as a cell detachment/attachment factor [22, 23]. This polymer has also been used in eye drop preparations and in neurosurgery as an embolic substance. In such cases, no* in vitro* cytotoxicity or acute toxicity in mice was noted [24–26].

The purpose of this study is to evaluate the ocular safety of pNIPAM, using clinical, electrophysiological, and histologic assessments, after intravitreal injections of excess quantities of pNIPAM adhesive preparations in the rabbit eye.

## 2. Methods

pNIPAM was synthesized through bulk polymerization of 1.0 g of N-isopropylacrylamide (NIPAM) with 0.0145 g of 2, 2-azobisisobutyronitrile (AIBN) in 14 mL of ethanol. The mixture was heated with an oil bath set at 65°C for 16 hours under constant stirring. After removal of ethanol under vacuum, the polymer was dissolved in a minimum amount of tetrahydrofuran and recrystalized by the addition of ether. The solid precipitates were collected and air dried. The solid pNIPAM was then dissolved in phosphate-buffered saline at a 50% concentration.

Studies were in compliance with the ARVO statement on the Use of Animals in Ophthalmic and Visual Research. After Institutional Animal Care and Use Committee and Institutional Review Board approval from the University of Southern California, twelve New Zealand pigmented rabbits weighing between 2 and 4 kg underwent intravitreal injections of pNIPAM. Preoperative pain control was achieved. The rabbits were anesthetized and the procedure was performed under sterile conditions. A 28-gauge needle was obliquely and transconjunctivaly inserted through the superior sclera, 1.5 mm behind the limbus. The needle was inserted into the vitreous under direct vitreous cavity view using a 65-diopter fundus lens. Once in the vitreous cavity, the syringe was directed so that the needle tip came to lie just anterior to the posterior retina. The bevel of the needle was directed toward the inferior retina, and 0.1 mL of the 50% tissue adhesive was injected. No leakage occurred at any time during the injection procedure.

At regular intervals, the rabbits underwent routine evaluations, including slit-lamp examinations, intraocular pressure measurement, indirect ophthalmoscopy, external and fundus color photography, fundus fluorescein angiography (FA), optical coherence tomography (OCT), and electroretinography (ERG). Slit-lamp examinations and indirect ophthalmoscopy were performed preoperatively, immediately after injection and on days 1, 2, 4, 7, and 14, and monthly thereafter for 6 months. Intraocular pressure was measured with a tonometer at each examination. FA and external and fundus photography were performed before operation, on day 14, and then on monthly basis for 6 months. ERG was recorded before operation, on day 14, and on monthly basis thereafter, using an Espion Visual Electrophysiology unit (Diagnosys LLC, Lowell, MA, USA) [27, 28].

After the last follow-up examination, the rabbits were sacrificed using an intracardiac injection of pentobarbital. Then, the eyes were enucleated, placed in Davidson fixative medium, and embedded in paraffin before sectioning. Histological study was performed in all study eyes. Tissue slices of 10 *μ*m from the frozen sections were cut until the sclerotomy location was reached and were stained with hematoxylin and eosin. Digital photographs from the histological slides were taken and studied using the NIH Image software (version 1.62, National Institutes of Health, Bethesda, MD).

## 3. Results

Following liquid pNIPAM intravitreal injection, glue polymerization occurred rapidly in the posterior vitreous; a filament of the glue also remained at the injection site as the needle was removed from the rabbit eye. Mechanical damage to the sclera and conjunctiva aside from at the injection sites themselves was not observed.

**Figure 1:**
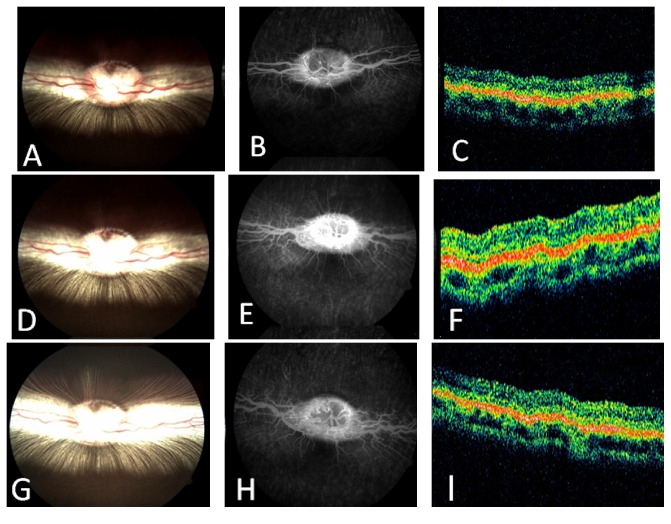
Color fundus photographs, fundus fluorescein angiography, and optical coherence tomography (OCT) from the baseline (A, B, and C), 3 months (D, E, and F), and 6 months follow-up (G, H, and I). There were no retinal or optic disc abnormalities in the study rabbits during the follow-up period.

**Figure 2:**
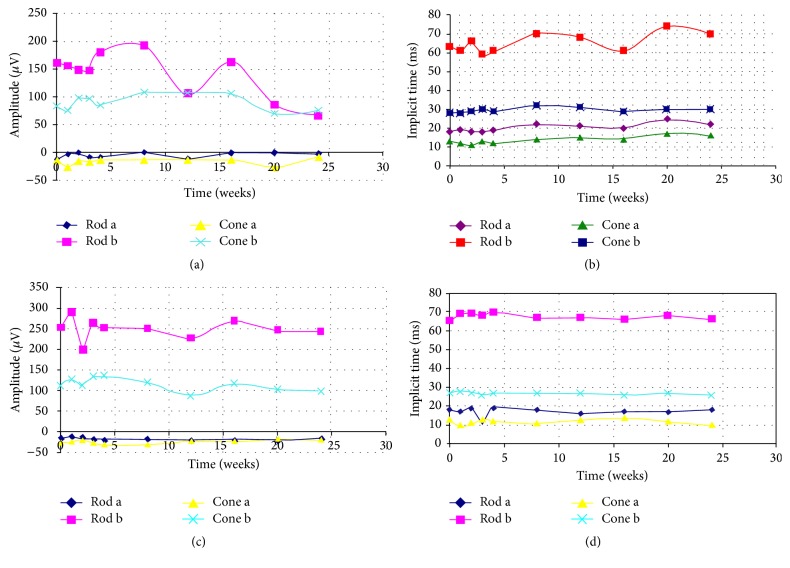
Mean b-wave amplitude response curves and implicit time responses at 6 months. (a and b) Eyes which underwent intravitreal injection of 0.1 mL of pNIPAM demonstrate a slight depression of scotopic and photopic responses when compared to respective control fellow eyes (c and d). Comparisons of the preoperative b-wave ratios versus the ratios at each time exam revealed no significant differences (*P* < 0.5) when calculated with the Student *t*-test.

All the animals showed mild to moderate chemosis and conjunctival swelling during the first month of follow-up. The amount of chemosis and conjunctival swelling gradually decreased and, at the third month of follow-up, minimal anterior segment inflammation was visible. The same level of inflammatory reaction was also observed in the anterior chamber and in the anterior vitreous during the first 7 follow-up days. After this period, the amount of the inflammatory reaction progressively decreased and completely disappeared after the third month of the follow-up period. Lens and cornea remained clear during the entire follow-up period. Intraocular pressure remained normal, ranging from 12 to 18 mmHg in the study eyes. Infection or fundus lesions, such as retinal thinning, retinal whitening, or chorioretinal adhesion around the optic disc and medullary wings, were not detected in any of the eyes during the follow-up period. OCT did not reveal retinal abnormalities such as hyperreflectivity of the outer retina or any chorioretinal thinning. Areas of retinal hypo/hyperfluorescence or neovascularization were not observed on FA in any of the rabbits (Figure 1). During the study follow-up, the mean b-wave amplitude ratios of electroretinogram recordings (eyes with pNIPAM injection/fellow eyes) remained close to 1 (Figure 2). Comparisons in pair of the preoperative b-wave ratios versus the ratios at each time exam revealed no significant differences (*P* < 0.5) when calculated with Student's *t*-test.

**Figure 3:**
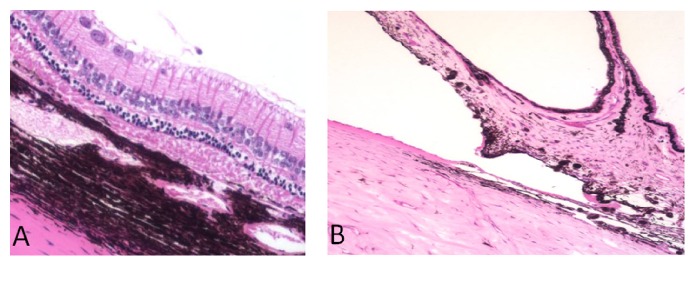
(A) Light microscopy of the retina 6 months after intravitreal injection of pNIPAM. The ganglion cell layer is facing upwards. Signs of retinal toxicity such as inner or outer retinal atrophy were not detected. (B) Light microscopy of the anterior chamber 6 months after intravitreal injection of pNIPAM. The iris tissue is facing upwards. The iris and the anterior chamber angle showed no abnormalities or damage.

The enucleated eyes were halved and the interior wounds grossly observed. All inside wounds were sealed and no vitreous incarceration or hemorrhage was detected at the sclerotomy locations. Light microscopy demonstrated an unremarkable anterior segment and the absence of cataract or intraocular inflammation in the sectioned eyes. There were no signs of retinal toxicity, such as inner or outer retinal atrophy, and the longitudinal sections of the optic nerve were unremarkable (Figure 3).

## 4. Discussion

A thermosensitive reversible adhesive could have several applications in the ophthalmology field, such as in intraocular drug delivery, posterior segment surgery, and implantation of biomimetic microelectronic devices. Preceding studies have shown that pNIPAM has a LCST of 32°C [29, 30]. This means that this thermosensitive hydrogel possesses decreased solubility in water as the temperature is raised due to a phase transformation at its LCST [29, 30]. Therefore, pNIPAM can be changed from a cellular detachment state to a cellular attachment state by changing the temperature of the tissue surface [29]. Due to its sharp property switch and the capability to transform the polymer into a solid state, pNIPAM could be categorized as a specialized chemical adhesive that may allow for applications that were formerly too complex or difficult to consider.

In our study, pNIPAM was injected intravitreally. The volume of pNIPAM injected was relatively large, especially when compared with the minute amounts delivered to the chorioretinal junction in both experimental models and surgical series of cyanoacrylate retinopexy for rhegmatogenous RD [31–33]. The excess quantity of pNIPAM did not cause any discernible distant ocular effects or localized retinal changes in the vicinity of the polymerized glue. There was no noticeable mechanical or chemical adverse reaction on the retina. Inflammatory reaction observed in the anterior chamber and the vitreous in the first days following the surgical procedure is a normal response observed in eyes that have undergone intravitreal injections. These effects, however, disappeared during the follow-up period.

The ideal adhesive for intraocular use should be safe, effective, and easy to use. In other words, it should be noninflammatory and nontoxic, possess a short curing time, have sufficient duration and strength of adhesion, and be easily deliverable. There is yet no standardized method to test the* in vitro* adhesive power of a material with the retina but a safe and effective adhesive could potentially be particularly useful in the posterior segment. In certain complicated cases of retinal detachment, an adhesive may obviate the need for internal tamponade, retinal laser photocoagulation, and face-down postoperative positioning. A retinal adhesive that achieves and maintains immediate closure of the hole, independent of body positioning, could ease the position and travel restrictions placed on these patients. Another potential application for intraocular adhesives would be in the attachment of retinal prostheses to the retina. Several groups around the world [34–37] are developing a retinal prosthetic device that will allow useful vision to be restored to patients with retinal degenerative diseases such as retinitis pigmentosa or age-related macular degeneration. Different methods have been proposed to attach this device to the retina, such as placing the device under the retina (a procedure that would not require array fixation) and the use of retinal tacks and different biocompatible glues [35, 36, 38].

Although N-isopropylacrylamide monomer is toxic to neural tissue, following polymerization, the pNIPAM molecule is no longer toxic to neural tissue and is commonly used in cell cultures and tissue cultures for its reversible cell adhesion properties [24, 29, 39]. pNIPAM has also been used in retinal pigment epithelium (RPE) cell cultures to provide RPE sheets for transplantation; these RPE cells preserved their morphology with no signs of toxicity [39]. Interestingly, pNIPAM has also been used to stop bleeding in experimental liver injuries and no toxicity has been reported [40]. To our knowledge there is no previous report on the* in vivo* effects of pNIPAM inside the eye.

In conclusion, intravitreal injections of pNIPAM in close proximity to retina showed limited intraocular inflammation without localized or diffuse retinal toxicity in the rabbit eye. pNIPAM proved to be safe for intravitreal injection in rabbits and if comparable safety is shown when applied to the retinal surface, it has the potential to be used for the management of retinal diseases such as rhegmatogenous RD, optic disc pit, and macular hole.

## Additional Points

*Summary Statement.* Intravitreal injections of poly-N-isopropylacrylamide in rabbits appear to be safe. Future studies are needed to evaluate the potential of pNIPAM as a bioadhesive in the treatment of retinal diseases.
